# Reduced predictable information in brain signals in autism spectrum disorder

**DOI:** 10.3389/fninf.2014.00009

**Published:** 2014-02-14

**Authors:** Carlos Gómez, Joseph T. Lizier, Michael Schaum, Patricia Wollstadt, Christine Grützner, Peter Uhlhaas, Christine M. Freitag, Sabine Schlitt, Sven Bölte, Roberto Hornero, Michael Wibral

**Affiliations:** ^1^Biomedical Engineering Group, E. T. S. Ingenieros de Telecomunicación, University of ValladolidValladolid, Spain; ^2^Commonwealth Scientific and Industrial Research Organisation, Computational InformaticsMarsfield, NSW, Australia; ^3^MEG Unit, Brain Imaging Center, Johann Wolfgang Goethe UniversityFrankfurt am Main, Germany; ^4^Department of Neurophysiology, Max-Planck Institute for Brain ResearchFrankfurt am Main, Germany; ^5^Institute of Neuroscience and Psychology, University of GlasgowGlasgow, UK; ^6^Department of Child and Adolescent Psychiatry, Psychosomatics and Psychotherapy, Johann Wolfgang Goethe UniversityFrankfurt am Main, Germany

**Keywords:** autism spectrum disorder, information theory, active information storage, complex systems, magnetoencephalography, hippocampus, predictive coding

## Abstract

Autism spectrum disorder (ASD) is a common developmental disorder characterized by communication difficulties and impaired social interaction. Recent results suggest altered brain dynamics as a potential cause of symptoms in ASD. Here, we aim to describe potential information-processing consequences of these alterations by measuring active information storage (AIS)—a key quantity in the theory of distributed computation in biological networks. AIS is defined as the mutual information between the past state of a process and its next measurement. It measures the amount of stored information that is used for computation of the next time step of a process. AIS is high for rich but predictable dynamics. We recorded magnetoencephalography (MEG) signals in 10 ASD patients and 14 matched control subjects in a visual task. After a beamformer source analysis, 12 task-relevant sources were obtained. For these sources, stationary baseline activity was analyzed using AIS. Our results showed a decrease of AIS values in the hippocampus of ASD patients in comparison with controls, meaning that brain signals in ASD were either less predictable, reduced in their dynamic richness or both. Our study suggests the usefulness of AIS to detect an abnormal type of dynamics in ASD. The observed changes in AIS are compatible with Bayesian theories of reduced use or precision of priors in ASD.

## 1. Introduction

It has been 70 years since Kanner (1943) and Asperger (1944) first described an intriguing disorder characterized by the children's inability to relate themselves in the ordinary way to people and situations from the beginning of life. The symptom cluster described by Kanner has been called autism spectrum disorder (ASD), and it is clinically defined by a triad of deficits comprising impairments in communication, social interaction, and behavioral flexibility (Wing and Gould, 1979). Prevalence studies estimate that ASD affects 2–10 children per 1000 births (Yeargin-Allsopp et al., 2003; Baird et al., 2006). It is characterized by an early onset, since these typical behaviors show up before the age of 36 months. Nevertheless, ASD is a permanent developmental disorder that will continue into adulthood. Great heterogeneity in development has been reported, with some individuals losing skills over time, others reaching a plateau in adolescence, and still others manifesting a pattern of continued development in adulthood (Seltzer et al., 2003). Due to the complexity and variety of the symptoms with which autistic individuals present to clinicians, it has been difficult to conceptualize a defining neurological mechanism that might underlie the core features of this disorder (Bauman and Kemper, 2005). Therefore, new techniques are necessary to achieve a more detailed understanding of this disorder, and ultimately an earlier identification and more effective interventions and treatment (Bauman and Kemper, 2005).

ASD symptoms, but also self-reports of ASD patients (Williams and Bishop, 1994) and the phenomenon of “savants” (Treffert, 2009) point to fundamentally altered modes of information processing in the brain of patients with autism. While autism has most likely genetic roots, the final disease outcome is the result of a developmental trajectory and of an interaction with the environment. It seems safe to assume that in the autistic brain information processing is also optimized or adapted during development in some way that genetics and environment allow, and the complex developmental trajectory of neuroanatomical changes in ASD supports this view (Bauman and Kemper, 2005). However, even describing this adapted, but altered information processing in ASD at a *neurophysiological* level—beyond behavioral outcomes—has remained difficult. Results at the neurophysiological level have so far mostly dealt with descriptors of the dynamics, such as time-frequency analysis (Sun et al., 2012), connectivity methods (Belmonte et al., 2004a,b) or entropy measures (Bosl et al., 2011). However, it has been difficult to address information processing more directly. These difficulties were foremost conceptual because what we actually mean when using the term *information processing* in biological systems has been unclear. Only recently formal, operational descriptions of information processing and its components have become available (Langton, 1990; Mitchell, 2011; Lizier, 2013). These descriptions can be traced back to Turing's finding that every act of information processing can be decomposed into the component processes of information storage, transfer and modification (Turing, 1936). Later, Langton and others expanded these concepts to describe the emergence of the capacity to perform arbitrary information processing algorithms, or “universal computation,” in complex systems, such as cellular automata (Langton, 1990; Mitchell, 2011).

Of the three component processes above—information transfer, storage, and modification—information storage in particular has been used with great success to evolve (Prokopenko et al., 2006), and optimize (Dasgupta et al., 2013) self-organizing information processing systems. This success was enabled by the introduction of quantitative measures of information storage in the form of excess entropy by Grassberger (1986) (introduced as “effective measure complexity,” and later reintroduced as excess entropy by Crutchfield and Feldman, 2003), and in the form of the *active information storage* (AIS) by Lizier et al. (2012). Despite their success in artificial systems, however, these measures have not been applied yet to biological neural systems.

One reason for this slow adoption may be that we face an apparent complication in biological neural systems, as in these systems information storage may at first sight take various forms, e.g., as reverberant neural activity or as synaptic changes (Zipser et al., 1993). However, a use of the stored information for information processing inevitably requires its re-expression in neural activity and its interaction with ongoing neural activity and incoming information. Hence, information storage actively in use for a computation will be reflected in the dynamics of neural activity, and is therefore accessible based on recordings of neural activity. Information storage in neural activity will be reflected by the fact that information from the past of a neural process will serve to predict a certain fraction of information in the future of this process, by virtue of the very definition of storage. To measure information-theoretically this amount of information in the future of a process that is predicted by its past state, we use AIS (Lizier et al., 2012), described in section 2.1.

It is via this predictable information that information storage is also tightly connected with predictive coding, an important family of theories of cortical function. Predictive coding theories propose that a neural system is constantly generating predictions about the incoming sensory input (Rao and Ballard, 1999; Friston et al., 2006; George and Hawkins, 2009; Bastos et al., 2012; Grossberg, 2013) to adapt internal behavior and processing accordingly. The prediction of incoming information that forms the central idea of predictive coding theory must happen via neural activity. These predictions typically need to be maintained for a short interval—as it is not known precisely *a priori* when the predictive information will be needed. Hence, the neural activity subserving prediction must itself have a predictable character, i.e., non-zero information storage. Analysis of AIS thereby enables us to test central assumptions of predictive coding theories rather directly.

The close link between information storage and general theories of cortical function makes AIS also a promising candidate measure to investigate altered information processing in ASD. Influential accounts of altered perception in ASD hold that there is either some form of reduced top–down control (Happé and Frith, 2006; Pellicano and Burr, 2012; Friston et al., 2013), or a reduced noise in the ascending sensory systems (e.g., Mottron et al., 2006). Both views can be formalized using a Bayesian formalism, i.e., a predictive coding theory of perception in ASD (Pellicano and Burr, 2012). Despite this semi-quantitative formalism, many aspects of altered perception in ASD can be explained in a Bayesian framework in one of two opposing ways—either by less dominant (top–down) expectations or more precise sensory inputs (Brock, 2012). Here, quantities such as the amount of predictable information in a neural signal—as measured by AIS—may play a crucial role in distinguishing these theoretical accounts based on experimental evidence, as they quantify the amount of information that is reliably obtainable from a brain area.

To explore the potential of AIS for ASD research, we here apply this measure to magnetoencephalographic (MEG) data obtained from a group of patients with high functioning ASD and matched healthy controls. We focus our study on the visual system, as atypical perception is particularly well documented in this system (see Williams and Bishop, 1994; Plaisted, 2001; Ropar and Mitchell, 2002; Mitchell and Ropar, 2004; Bertone et al., 2005; Rogers and Ozonoff, 2005; Happé and Frith, 2006; Mottron et al., 2006; Sheppard et al., 2007; Baron-Cohen et al., 2009; David et al., 2010, but also see, Ropar and Mitchell, 1999, 2001).

## 2. Materials and methods

### 2.1. Active information storage—definition and practical estimation

We assume that the neural signals we record from a system 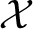 can be treated as realizations *x*_*t*_ of random variables *X*_*t*_ that together form the random process X, describing the system's dynamics.

AIS is then simply defined as the mutual information $I\left(X_{t\;-\;1}^{k-};X_{t}\right)$—see Cover and Thomas (1991)—between the past state random variable **X**^*k*−^_*t* − 1_ = {*X*_*t* − 1_, …, *X*_*t* − 1 − *k*_} of a process and its next random variable *X*_*t*_ (Lizier et al., 2012):

(1) $$A_{X_{t}}=\underset{k\to \infty }{\lim}I\text{​}\left(X_{t\;-\;1}^{k-};X_{t}\right)=\underset{k\to \infty }{\lim}\langle \log\frac{p\left(x_{t\;-\;1}^{k-},x_{t}\right)}{p\left(x_{t\;-\;1}^{k-}\right)p\left(x_{t}\right)}\rangle \text{​}.$$

Here the averaging 〈·〉 via $p\text{ ​}\left(x_{t\;-\;1}^{k-},x_{t}\right)$ in principle has to be taken over an ensemble of realizations of the process at time point *t* (e.g., via physical replications of the system 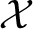). For stationary processes, however, where all random variables that form the process X have identical probability distribution, we can use time-averaging instead of the ensemble average and Equation (1) simplifies to:

(2) $$A_{X}=\underset{k\to \infty }{\lim}{\langle \log\frac{p\left(x_{t\;-\;1}^{k-},x_{t}\right)}{p\left(x_{t\;-\;1}^{k-}\right)p\left(x_{t}\right)}\rangle }_{t}$$

where the averaging can now be taken with respect to time *t*.

The use of the multivariate collection **X**^*k*−^_*t* − 1_ is particularly important here—it is intended to capture the *state* of the underlying dynamical system 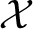, and can be viewed as a state-space reconstruction of it. In this fashion, AIS brings together aspects of both dynamical systems theory and information theory in its analysis. The AIS tells us how much information could be *predicted* about the next measurement of a process by examining its past state. For a linear perspective, this is akin to building a classical autoregressive model of order *k* and measuring how well that model predicts the next measurements of the process. Importantly though, the use of information theory here is a more general approach which captures non-linear auto-dependencies in the process, and does so in a model-free way. As such, we refer to this component of the prediction of the next measurement as *information storage*, capturing the information-theoretic basis of the self-prediction. This also highlights that AIS quantifies how much information from the past state is involved in generating or computing the next value of a process, in contrast to other information sources (i.e., information transferred from other processes as quantified by the transfer entropy Schreiber, 2000, a non-linear analogy of the Granger causality) as discussed by Lizier et al. (2010). We call this the *active* component of information storage since it quantifies the stored information actively *in use* in this generation of the next value, as opposed to that passively stored for later use, e.g., in synaptic weights. Zipser et al. (1993) discuss this contrast in active and passive storage, though our perspective generalizes the active storage beyond merely “maintaining neural activity” (as described by Zipser et al., 1993) to more complex non-linear auto-correlations, and may additionally capture contributions of passive storage when they are re-expressed in dynamics.

Now, if the history before a certain time point *t* − *k*_max_ does not help to improve the prediction of *X*_*t*_ we can further simplify Equation (2). Technically speaking, *X*_*t*_ then is conditionally independent of all *X*_*t*−*k*_*i*__ with *k*_*i*_ > *k*_max_:

(3) $$\underset{k\to \infty }{\lim}p\text{ ​}\left(x_{t}|x_{t\;-\;1}^{k-}\right)=p\text{ ​}\left(x_{t}|x_{t\;-\;1}^{k_{\text{max}}-}\right)\text{​},$$

and Equation (2) becomes:

(4) $$A_{X}={\langle \log\frac{p\left(x_{t\;-\;1}^{k_{\text{max}}-},x_{t}\right)}{p\left(x_{t\;-\;1}^{k_{\text{max}}-}\right)p\left(x_{t}\right)}\rangle }_{t}\text{​​}.$$

The parameter *k*_max_ can be determined using Ragwitz' criterion (Ragwitz and Kantz, 2002), as suggested for example in Vicente et al. (2011), and implemented in the TRENTOOL toolbox (Lindner et al., 2011). For the analyses presented here, we used *k*_max_ = 10 on data with a sampling rate of 300 Hz.

For the practical estimation of Equation (4) for continuous data, as analyzed here, various estimation techniques exist, such as binning and kernel approaches. Here, we used a kernel-based estimator (see Kantz and Schreiber, 2003 for more information on kernel-based estimators) as implemented in the open source JAVA Information Dynamics Toolkit (Lizier, 2012), with a kernel width ϵ of 0.5 standard deviations of the data.

### 2.2. Data acquisition

We recorded magnetoencephalography (MEG) signals in 10 ASD patients and 14 matched healthy control (HC) subjects in a visual task. More details of this study can be found in Sun et al. (2012). Its most important aspects are summarized in the following paragraphs.

#### 2.2.1. Participants and task

All ASD patients (mean age: 30.3 ± 9.6) were clinically diagnosed and suffered from Asperger's disorder, or pervasive developmental disorder not otherwise specified (PDD-NOS) according to DSM-IV (American Psychiatric Association, 2000). The clinical diagnosis was corroborated using the German form of the Autism Diagnostic Interview-Revised (Schmötzer et al., 1993; Lord et al., 1994) and the Autism Diagnostic Observation Schedule (Lord et al., 2000). The patients were recruited from the Department of Child and Adolescent Psychiatry, Psychosomatics, and Psychotherapy of the Goethe University at Frankfurt/M. The healthy controls (mean age: 29.7 ± 6.9) were screened for psychopathology with the German version of Structured Clinical Interview for DSM-IV-R Non-Patient Edition (Saß et al., 2003). Both groups showed no significant differences in age, sex distribution and IQ. The study was performed according to the Declaration of Helsinki and approved by the ethics committee of the Goethe University (Frankfurt, Germany).

All subjects performed a perceptual closure task where stimuli consisted of degraded pictures of human faces in which all shades of gray had been converted into black or white (Mooney and Ferguson, 1951). In addition, scrambled and vertically mirrored versions of these stimuli were created, for which face perception was not possible. One hundred and sixty different stimuli for each stimulus category were presented in a random sequence, where each stimulus was shown for 200 ms, separated by a random inter-stimulus intervals between 3500 and 4500 ms. Participants had to indicate with a button press whether they saw a face or not. Response hands were counterbalanced across participants in each group. This set of stimuli allowed us to identify the visual system and higher cortices related to object and face perception for further investigation using AIS.

#### 2.2.2. MR and MEG data acquisition

Individual structural MR images were acquired with a Siemens Allegra scanner (Siemens Medical Solutions, Erlangen, Germany), using a 3D MPRAGE sequence. MEG signals were recorded with a 275-channel system (Omega 2005; VSM MedTech, Coquitlam, BC, Canada) with 600 Hz sampling rate, third-order gradiometers. The acquired data were bandpass filtered between 0.5 and 150 Hz (fourth order Butterworth filter). Before and after each run, the head position was localized using localization coils. Recordings with movements larger than 5 mm were discarded.

#### 2.2.3. MEG data preprocessing

MEG data were analyzed using the FieldTrip open source MATLAB Toolbox (Oostenveld et al., 2011). The continuously recorded data were segmented into trials from −1000 to 1000 ms with respect to the onset of the visual stimulus. Eye blinks, signal jumps caused by the SQUID sensors, and muscle artefacts were detected automatically (in this sequence) using the preprocessing functions of FieldTrip, followed by visual inspection for residual artefacts. Affected trials were rejected completely as suggested in Gross et al. (2012). The remaining trials were linearly detrended and baseline corrected.

#### 2.2.4. Analysis of sensor-level spectral power changes

Time-frequency representations (TFRs) were computed from sensor data using a multi-taper method [frequency range from 25 to 140 Hz in 2 Hz steps over a time range of −500 to 1000 ms in 10 ms steps, discrete prolate spheroidal sequences (DPSS), length of sliding time window, 5/frequency, width of frequency smoothing, 0.4 * frequency]. The power of the time-frequency-transformed trial data was averaged over all sensors and trials and, subsequently, all subjects. The optimal beamformer bandwidth (Brookes et al., 2008) was then estimated based on the observed power changes induced by the visual stimulus (analysis interval 75–375 ms) relative to baseline (analysis interval −350 to −50 ms).

#### 2.2.5. Source reconstruction and selection

As the estimation of AIS is computationally very demanding, we were not able to compute this measure on a source grid covering the whole brain. We therefore chose to investigate a selection of source locations showing differences between baseline and the perceptual closure task. This decision was based on previous reports of changes in visual perception in ASD (see Pellicano and Burr, 2012 and references therein); this goal for a selection of sources substantially differs from the goal of detecting sources with spectral power differences between ASD subjects and controls that was pursued in the study by Sun et al. (2012), and the analysis strategies differ accordingly. Note that preselecting sources with power differences between ASD and healthy controls would potentially bias a subsequent analysis of ASD (“double dipping”), whereas selecting areas that represent the visual system does not entail such a bias *a priori*. To identify visually responsive areas, we first performed a beamformer source analysis in the high gamma frequency range (60–120 Hz), as the initial TFR-analysis indicated sustained responses triggered by the visual stimulus in this frequency range. Note that even though differences between ASD patients and healthy controls have been demonstrated previously in this band, the choice of this band does not unduly bias the analysis as such differences have been shown in all major frequency bands (see Figure 3 in Sun et al., 2012).

After identifying locations with significant differences in the high-frequency gamma band, we then recomputed broadband beamformer filters for these locations and extracted the individual source time courses for each subject and source location for further analysis. Note that we only used baseline intervals (time interval from −1000 to 0 ms with respect to stimulus onset) in our analysis of AIS to ensure the stationarity of the underlying processes. Note that differences in brain dynamics are also expected in this baseline interval, as there is of course ongoing visual experience.

In more detail, we constructed head models of each individual subject from anatomical MRI for beamformer source reconstruction. To this end, first a regular source grid with a spacing of 1 cm was constructed in MNI space. After computing the (linear) transformations from the MNI template head to each individual subject's anatomical MRI, these transformations were applied to the source grid to obtain individual source grids in physical space for each subject. After segmentation of the MRI to find the inner boundary of the skull, the lead fields for the individual source grid locations were then computed using a realistic single shell model introduced by Nolte (2003).

Next, the cross spectral density (CSD) matrices were computed for all trials for both patients and controls, separately for baseline (−350 to −50 ms) and task intervals (75–375 ms) in the high (60–120 Hz) gamma-band frequency range, using a multi-taper method (center frequency 90 Hz, smoothing bandwidth ±30 Hz, DPSS, 17 tapers). Based on the lead fields and the computed CSD matrices, spatial filters were computed for each grid point using a frequency domain beamformer (Gross et al., 2001) as provided by FieldTrip, using real valued filter coefficients. To compensate for the rather short time intervals underlying the computation of CSD matrices, a matrix regularization of λ= 5% of the trace of the CSD matrix was used. In order to avoid that statistical differences arise because of different filters for the two intervals, we computed common filters which are based on the combined CSD matrices from both, task segments (face and no-faces) and the corresponding baseline segments (Nieuwenhuis et al., 2008; Gross et al., 2012). The source power estimate at each grid point was computed by applying the corresponding common filter of this grid point to the filtered trial data. This was done separately for task and baseline segments of each subject.

To obtain common sources that responded to the perceptual closure task for all subjects and independent of their group affiliation, a non-parametric randomization test (test-statistic: cluster sum of dependent samples *t*-metric, Monte Carlo estimate with 5000 randomizations, Maris and Oostenveld, 2007) was computed based on source power data of all subjects. Using a within-subject design on subject-wise source power for task and baseline, activation-versus-baseline effects were identified; *t*-metrics within a cluster were used to identify local extrema of source power changes inside the significant clusters.

### 2.3. Analysis of active information storage in source time courses

For further analysis of AIS based on source time courses, we obtained these time courses at the identified source locations that responded to the perceptual closure task, using a broadband beamformer, so that AIS computation could draw on a signal bandwidth of 10–150 Hz—the analysis of AIS at even lower frequencies was not possible due to the finite length (1 s) of the baseline data. On the three source time courses extracted for the three cardinal spatial directions (*x, y, z*) at each location we then performed a principal component analysis in order to determine the dominant dipole orientation (direction with the largest variance), and kept only the signal for this direction. As indicated above, AIS was computed using the Java Information Dynamics Toolkit (Lizier, 2012), with a box kernel of a width of 0.5 standard deviations of the data, and a history length *k* of 10 time steps. Per subject and source, approximately 40.000 samples entered the AIS analysis, composed of 1 s of baseline data per trial, sampled at 300 Hz, repeated across approximately 66 correct and artefact-free trials per condition, and two conditions. For statistical comparison between ASD patients and controls we used a randomization test, and corrected the significance threshold for multiple comparisons across 12 sources using the false discovery rate (FDR) with a threshold of *q* < 0.1, as suggested in Genovese et al. (2002).

### 2.4. Correlation of band-limited spectral power, autocorrelation decay time, and active information storage

To investigate whether the obtained AIS values were driven by spectral power changes, or contained information not accessible by an analysis of spectral power, we computed the correlation coefficients between spectral power in the 10–12 Hz α-, the 13–15 Hz β-, the 25–60 Hz low frequency γ-, and the 60–120 Hz high-frequency γ-bands. Spectral power within these bands was computed per subject using a Hanning window on the full baseline data followed by a fast Fourier transform for frequencies up to 25 Hz. Above 25 Hz spectral power was determined by a multitaper approach using 34, and 59 DPSS-tapers for the bands from 25 to 60 Hz and from 60 to 120 Hz, respectively.

In addition, we determined the autocorrelation decay time (ACT) as a measure of linear memory time scales in the data. The ACT was obtained by computing the autocorrelation function and determining the lag at which the autocorrelation function had dropped to a fraction of 1/*e* of its center peak.

## 3. Results

### 3.1. Sources participating in the perceptual closure task

**Figure 1:**
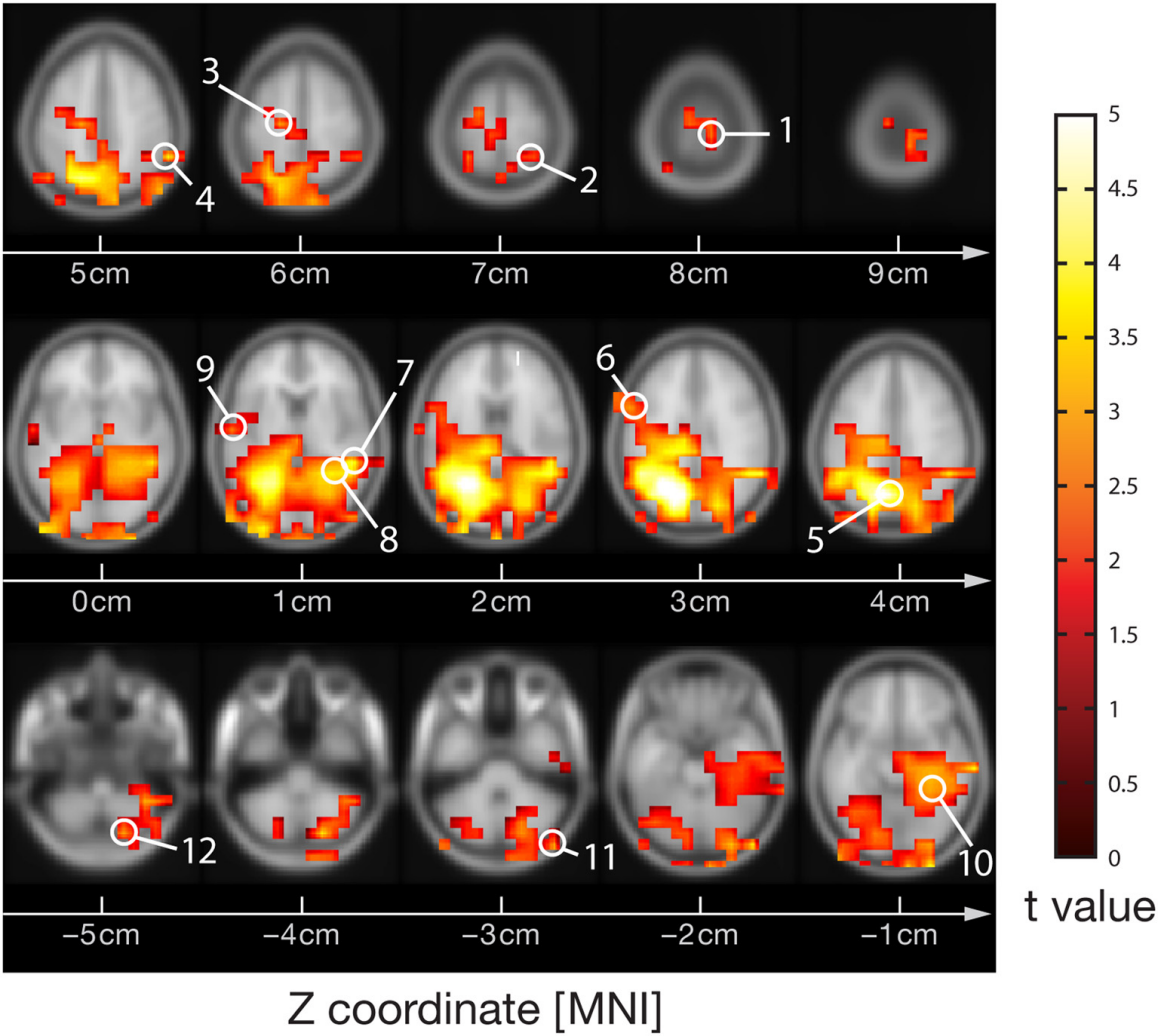
**MEG-beamformer source locations used for the analysis of active information storage**. MEG sources with enhanced power in the high-frequency gamma band (60–120 Hz) upon visual stimulation with Mooney face images (see Grützner et al., 2010 for stimulus details); permutation test on *t*-metrics *p* < 0.05, cluster-based correction for multiple comparisons. Source locations: 1—Primary motor cortex BA4a R (10, −30, 80), 2—Superior parietal lobule 7PC R (30, −50, 70), 3—Premotor cortex BA6 L (−20, −20, 60), 4—Parietal lobe (60, −50, 50), 5—Precuneus/Superior parietal lobule 7P L (−10, −70, 40), 6—Broca's area BA44 L (−60, 10, 30), 7—Temporal lobe (50, −40, 10), 8—Visual cortex V2 BA18 R/V1 BA17 R (30, −50, 10), 9—Secondary somatosensory cortex/Parietal operculum OP4 L (−60, −10, 10), 10—Hippocampus/Subiculum R (30, −40, −10), 11—Right cerebellum (50, −90, −30), 12—Cerebellum (20, −80, −50)

Visual stimulation increased neural activity in the high-frequency gamma band in several occipital, parietal, temporal and central cortical regions and in the cerebellum (*p* < 0.05, corrected) (Figure 1), as expected from previous studies (Grützner et al., 2010; Sun et al., 2012). Source locations of significantly increased gamma-band power were: 1—Primary motor cortex BA4a R (10, −30, 80), 2—Superior parietal lobule 7PC R (30, −50, 70), 3—Premotor cortex BA6 L (−20, −20, 60), 4—Parietal lobe (60, −50, 50), 5—Precuneus/Superior parietal lobule 7P L (−10, −70, 40), 6—Broca's area BA44 L (−60, 10, 30), 7—Temporal lobe (50, −40, 10), 8—Visual cortex V2 BA18 R/V1 BA17 R (30, −50, 10), 9—Secondary somatosensory cortex/Parietal operculum OP4 L (−60, −10, 10), 10—Hippocampus/Subiculum R (30, −40, −10), 11—Right cerebellum (50, −90, −30), 12—Cerebellum (20, −80, −50). For these locations broadband beamformer source time-courses for the baseline interval were extracted and subjected to AIS-analysis (recall that only baseline interval values were analyzed to ensure stationarity of the data).

### 3.2. Active information storage

**Figure 2:**
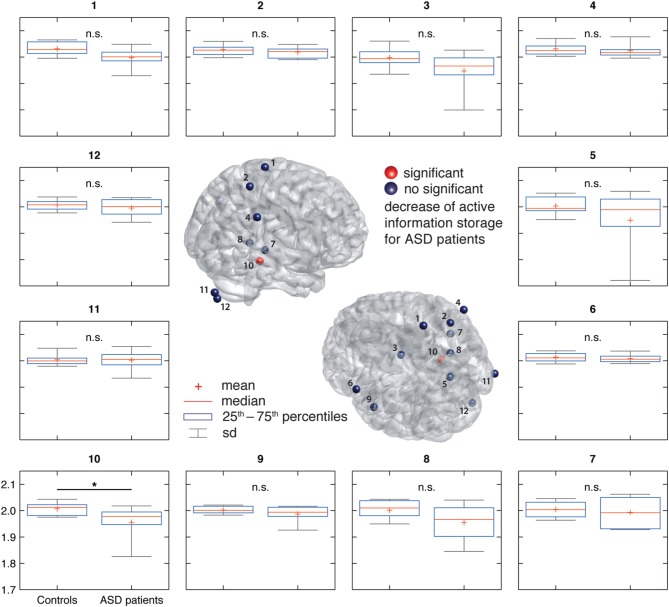
**Active information storage (AIS) results in ASD and control groups**. (Center) Source locations (spheres) overlaid on a standard MNI brain. Sources are colored red or blue to indicate significant or non-significant differences between groups, respectively. (Surround) Box and whisker plots for the data of each investigated source as indicated by the number in the subfigure's title. For source locations see Figure 1.

AIS-analysis revealed significantly reduced AIS in ASD in the hippocampus (*q* < 0.1, FDR corrected) (Figure 2). At an uncorrected significance level (*p* < 0.05), we observed additional differences in visual cortex, primary motor cortex and premotor cortex. At a purely descriptive level, in 11 out of 12 sources the observed median AIS values were lower in the ASD group compared to controls.

### 3.3. Correlation of spectral power, autocorrelation decay time, and AIS

**Figure 3:**
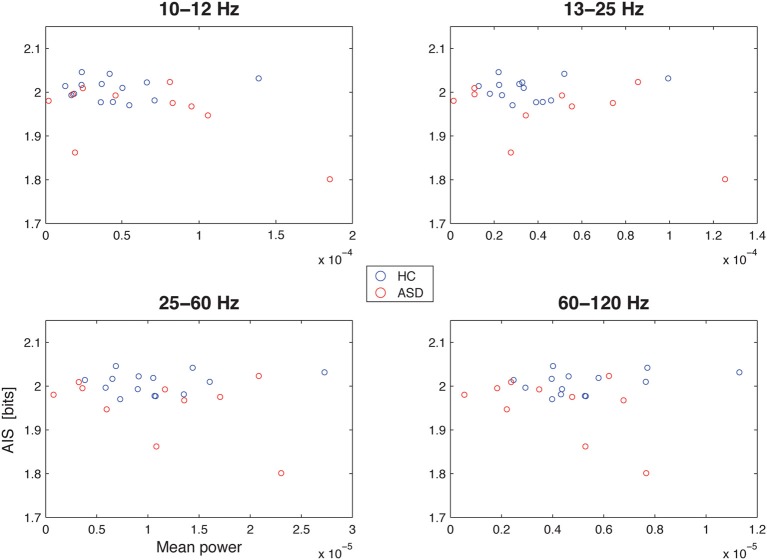
**Correlation between spectral power and AIS in the hippocampal source**. Correlation between spectral power in the 10–12 Hz α-, the 13–15 Hz β-, the 25–60 Hz low frequency γ-, and the 60–120 Hz high-frequency γ-bands (x-axes) and the active information storage (y-axes). See Table 1 for details on correlation coefficients.

Spectral power in none of the investigated bands (10–12, 13–15, 25–60, and 60–120 Hz) was significantly correlated with AIS values after correction for multiple comparisons (Table 1)(minimum *p*-value reached by any correlation: *p* = 0.03, uncorrected), indicating that AIS provides information that is at least partially independent of spectral power indices (Figure 3). Moreover, correlation coefficients were mostly negative—in contrast to what would be expected of the bias properties of the AIS estimator (see the companion paper on *local* AIS in this *Frontiers special topic* Wibral et al., 2014 for details), further supporting the independence of the two measures for our data.

**Table 1:** **Correlation coefficients between AIS and spectral power, and autocorrelation decay time (ACT) in the hippocampal source**.

**Frequency (Hz)**	**Spearman**		**Pearson**	
	**ρ**	***p***	**ρ**	***p***
10–12	−0.202	0.343	−0.444	0.030
13–25	−0.143	0.502	−0.364	0.080
25–60	−0.034	0.876	−0.194	0.364
60–120	0.117	0.586	−0.048	0.825
ACT	−0.318	0.130	−0.434	0.034

**Figure 4:**
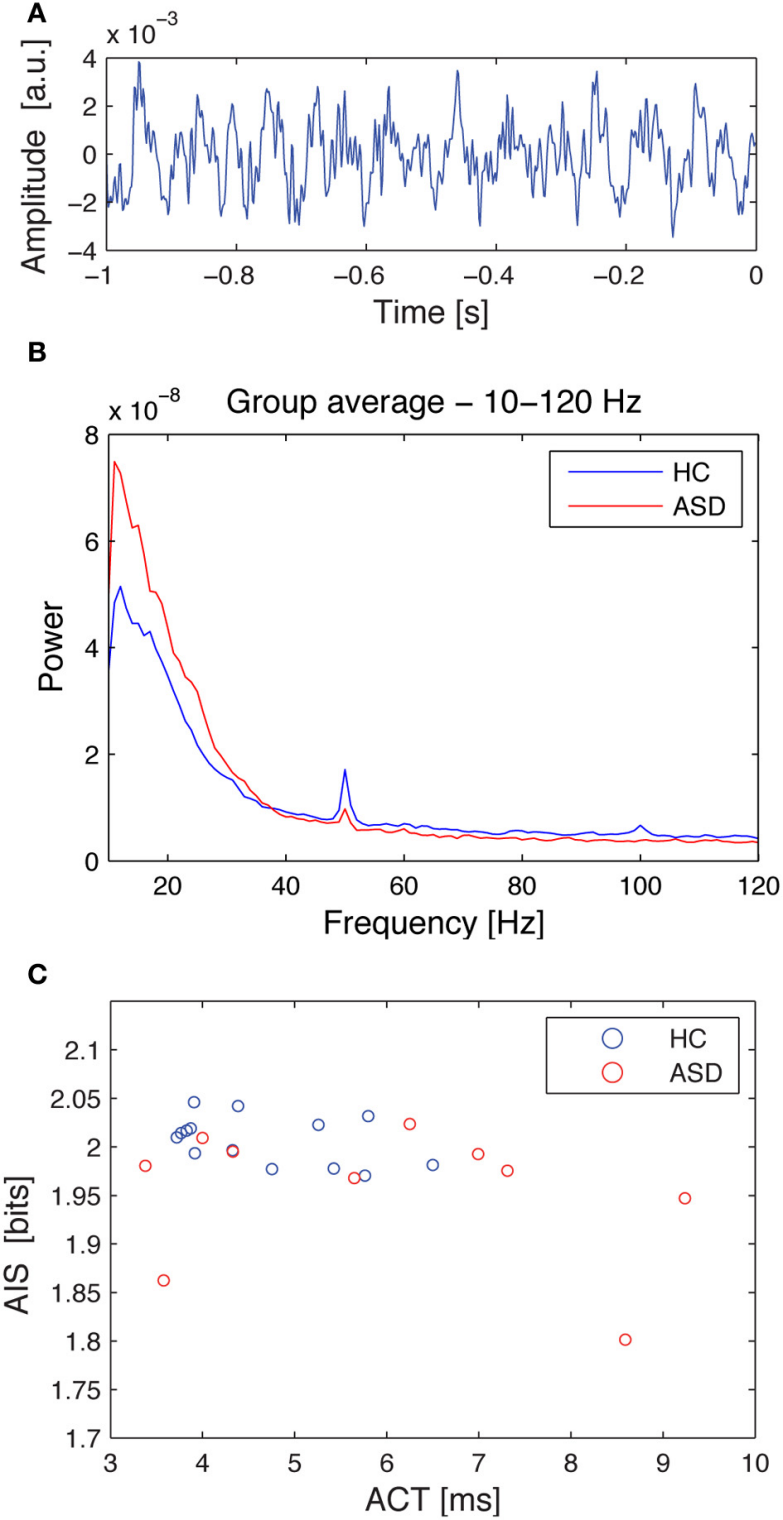
**Source time courses, power spectra and the correlation of autocorrelation decay constant, and AIS for the hippocampal source**. **(A)** Exemplary dipole moment time course of a single trial (baseline) for the hippocampal source. **(B)** Source spectral power for the hippocampal source, separately averaged for the healthy controls (HC, blue), and the ASD patients (ASD, red). Note that the spectrum was cut at 10 Hz as this was the lowest frequency included in the AIS analysis. **(C)** Correlation plot between the autocorrelation decay time (ACT) and the AIS. Data are shown separately for healthy controls (HC, blue), and the ASD patients (ASD, red). See Table 1 for details on correlation coefficients.

Similarly, ACT showed a negative Pearson correlation with the AIS (*p* < 0.034) (Figure 4).

## 4. Discussion

In line with our initial hypothesis, we found reduced AIS in individuals with ASD. More specifically, AIS was reduced in the hippocampus (subiculum). As our study is the first of its kind, extra care has to be taken to ensure both, an understanding meaning of the results at the conceptual level, as well as a clear view of the limitations of the current study. Therefore, we start by discussing two technical points related to a proper interpretation of AIS; first, we clarify the relation between AIS and signal prediction errors; second, we discuss the relationship of AIS and more high level concepts of memory in a neural system. Next, we detail the limitations of the current study in terms of small sample size and region of interest analysis. After these technical points, we discuss our findings in relation to known anatomical and cellular changes in ASD. We close by discussing our finding in relation to predictive coding theories of this disorder.

### 4.1. Active information storage and signal prediction errors

In the introduction, we pointed out how measures of information storage may be useful tools to investigate predictive-coding type of theories of cortical function (Rao and Ballard, 1999; Friston et al., 2006; George and Hawkins, 2009; Bastos et al., 2012; Grossberg, 2013). In this respect, it seems important to stress the difference between the amount of *predicted information*—as measured by information storage—and the *signal* prediction error, i.e., the amount of information not predicted in a signal (not to be confused with a neural prediction error in predictive coding theories). While the sum of these is the total information in a process, this total information is not necessarily constant. In fact, in most task-related studies we expect the neural processes to be non-stationary, i.e., to have probability distributions changing across time, leading to changing total information. This, in turn, results in predicted information (information storage) and unpredicted information (prediction error) describing complementary aspects of the information processing system—and one cannot be obtained from the other.

### 4.2. Active information storage, memory, and neural processing

While seemingly similar, AIS as an information theoretic measure should not be confused with high-level concepts of memory, or the storage of information about the external world. Rather, it describes the predictability and complexity of a neural process. AIS is low for processes that produce little information, such as a constant process, but also for unpredictable processes, such as chaotic ones (Lizier et al., 2011). Only when sufficiently rich dynamics and predictability meet, a high AIS value is obtained. In the context of our data, high AIS values are linked to transitions in the dynamics that are repeatedly seen across the multiple trials used for analysis—albeit not necessarily at the same time. Therefore, one source for reduced AIS values in the ASD group could be a more erratic signal behavior across trials in baseline dynamics between the stimuli. With respect to this baseline dynamics it is important to note that the baseline activity is not necessarily independent of stimuli and task. In this respect, the reduced AIS in the baseline epochs in ASD could still be linked to the specific stimulus material used here (faces, a social stimulus) and the detection task. In how far our results can be generalized for other experimental designs is an open question.

Correlation analysis between spectral power and AIS values revealed that AIS provides additional information, not immediately accessible using an analysis of spectral power. In contrast, the significant Pearson correlation between ACT and AIS indicates that the AIS reflects also the linear memory in the process, as would be expected. However, the correlation coefficient was below 0.5, indicating that also for the comparison of ACT and AIS, AIS yields additional useful information.

### 4.3. Reduced active information storage in ASD

Our results showed that AIS values were reduced in the neural signals obtained from the hippocampus/subiculum. Other sources showed reduction at least at an uncorrected significance level (visual cortex, primary motor cortex, premotor cortex). Before we proceed to the potential implications of these findings, we will briefly discuss several reasons that warrant a cautious interpretation of our results.

#### 4.3.1. Limited sample size

Perhaps the most important reason for caution is the relatively low number of patients in this study (*n* = 10), limiting statistical power. Therefore, our study should be understood as a pilot study for a larger, normative study of AIS values in ASD. Such a larger scale study seems highly promising as a close inspection of Figure 2 reveals that the mean and median AIS values in the ASD group are lower in *all* investigated sources, except one (right cerebellum). Despite the fact that none of these effects reaches statistical significance, the relatively uniform sign of the effect may point to a more pervasive reduction of AIS in ASD. This, however, can only be tested in a study with improved statistical power.

#### 4.3.2. Region of interest analysis and magnetoencephalographic detection of deep sources

Our focus on selected brain areas limits any statements on the ubiquity of reduced AIS in ASD. The fact that we focus on preselected brain areas (for purely practical reasons), clearly forbids any statements of the type “AIS is most strongly reduced in brain area A” or “AIS is only reduced in brain area A.” Thus, we can only link the current findings to literature on ASD-related changes in the specific brain areas that were investigated here. Furthermore, MEG source reconstruction is of limited spatial precision. We therefore discuss our findings of reduced AIS in the hippocampus/subiculum—where the analyzed source was located—more broadly as reduced AIS in the hippocampal region, and note the possibility of signal leakage from the nearby amygdala (note, however, that the cellular organization of the amygdala makes detectable MEG signals less likely to be picked up). With respect to the hippocampus it is often questioned whether the sensitivity of MEG recordings is high enough to capture this relatively deep source. However, a large body of evidence has accumulated in recent years that confirms that hippocampal activity can be reconstructed with modern MEG devices, artefact suppression techniques, and beamformer source reconstruction (Tesche et al., 1996; De Araújo et al., 2002; Hanlon et al., 2005; Cornwell et al., 2008; Riggs et al., 2009; Taylor et al., 2012). Moreover, nearby inferior temporal brain areas are routinely localized using MEG, for example the fusiform face area (Grützner et al., 2010); in addition, traces of thalamic activity have been recently revealed, using a combination of MEG cross-frequency analyses and transfer entropy techniques (Roux et al., 2013), and even auditory brain stem responses have been localized using MEG (Parkkonen et al., 2009). We therefore think it is safe to assume that our results indeed derive from changes in activity patterns in the hippocampal region.

### 4.4. The hippocampus in ASD

Interestingly, there is a number of anatomical findings of atypical hippocampal structure in patients with ASD. At the cellular level, increased cell packing density and reduced cell size was reported by Bauman and Kemper (1994). Raymond et al. (1995) further showed reduced dendritic branching of CA4 and CA1 cells. Blatt et al. (2001) reported reduced binding of GABAa receptors in the hippocampus. Furthermore, some rare Autism-linked point mutations coding for to Neuroligins seem to selectively target AMPA receptor-mediated neurotransmission in Hippocampus and dramatically change synaptic function in a mouse model of autism (Etherton et al., 2011).

At the macroscopic level, an enlargement of the right hippocampus was found across all studied age groups by Schumann et al. (2004), and an enlargement of the left hippocampus by Groen et al. (2010), Dager et al. (2007), and Nicolson et al. (2006) also reported shape abnormalities of the hippocampus in ASD. In addition, metabolic abnormalities in the hippocampus-amygdala region (Otsuka et al., 1999) have been reported as well.

Taken together, these structural and cellular findings suggest an involvement of hippocampal changes in the pathophysiology of ASD. Our findings are compatible with this idea and add a computational perspective to the neuroanatomical and cellular evidence by indicating that brain signals from hippocampus have less predictable information in patients with ASD. Next, we will discuss how these findings tie in with predictive coding accounts of ASD.

### 4.5. Reduced AIS and predictive coding theories of ASD

Given that the hippocampus is a plausible locus for changes in information processing in ASD from an anatomical perspective—how does the observation of reduced AIS in this brain area fit the various theoretical accounts of information processing in ASD?

For a more detailed understanding of the meaning of a reduction in AIS in relation to ASD, we have to consider first that reductions in AIS may indicate various changes in cortical dynamics—either a reduced dynamic richness of the neural process captured in the measurement, a decrease of predictability, or a combination of the two. Irrespective of the exact underlying change in the dynamics, however, a brain area receiving signals from another one with reduced AIS will inevitably be faced with a signal that has less predictable information. We can therefore state that information from cortical signals from one brain area will be harder to predict internally by another brain area in ASD patients.

If we look at this reduction in predictable information in ASD from the perspective of predictive coding theory, we may speculate that this reduction will result in difficulties in learning internal predictive models and in a less accurate model of the external world. Taking into account additionally that internal models should be organized hierarchically with internal models in sensory areas lowest in the hierarchy, as suggested for example by the hierarchical temporal model of George and Hawkins (2009), and by the model of Kiebel et al. (2008), the fact that we observe the most significant differences in hippocampus is particularly interesting. This is because the hippocampus resides at a high level in these hierarchies (George and Hawkins, 2009), where it would be most vulnerable to difficulties in learning of internal models. Moreover, many anatomical, physiological and computational reasons suggest that deeper or more central models at higher levels of the hierarchy entail dynamics that have greater temporal depth (Kiebel et al., 2008), and therefore should have more predictable information. One may speculate that this adds to the visibility of changes in AIS deep into the hierarchy, e.g., in hippocampus.

The fact that we obtained significantly reduced AIS values specifically in the *hippocampus* is also remarkable in relation to previous whole brain analyses of neuronal responses to explicit manipulations of predictability—using temporal dependencies in sequences of stimuli. For example, Strange et al. (2005) report hippocampal selectivity for the predictability of stimuli, consistent with the notion that the hippocampus is of central importance in the processing of temporal succession (MacDonald et al., 2011). This processing of a temporal aspect of predictability in hippocampus fits comfortably with the hierarchical Bayesian inference and predictive coding formulations of autism, when combined with the changes in hippocampal processing reported here. In other words, if altered hippocampal processing lead to a loss of hierarchically deep encoding of hidden causes in the world, this would necessarily entail a loss of deep temporal structure and a failure to encode temporal regularities over extended periods of time, and thereby global temporal context.

Such a loss of central or deep coherence in time and space has also been proposed previously as a psychological mechanism that explains many of the symptoms in ASD (“weak central coherence theory,” Frith, 1989, but see Bernardino et al., 2012 for conflicting data). This view indeed pre-dates modern perspectives from the point of view of hierarchical inference and predictive coding (Pellicano and Burr, 2012), but is fully compatible with its successors.

In sum, our data are fully compatible with predictive coding accounts of ASD (e.g., Pellicano and Burr, 2012). In contrast, our data are not compatible with theories that suggest enhanced sensory representations, and/or lower physiological noise, as both of these should lead to increased rather than reduced AIS values, which was not observed. Thus, our data favor accounts of autism in terms of compromised top–down processing.

### 4.6. AIS and previous studies on signal-entropy and complexity in ASD

Several previous studies have analyzed brain activity in ASD by means of complexity and/or entropy measures. For instance, Catarino et al. (2011) analyzed EEG data using multi-scale entropy, which quantifies the complexity of a physiological signal by measuring entropy across multiple time scales. Their results demonstrated a complexity reduction in autism group in comparison with controls, especially over tempo-parietal and occipital sensors. Using a modified version of multi-scale entropy, a decrease in resting-state EEG complexity in children at high risk for ASD has been reported (Bosl et al., 2011). In another study, Ahmadlou et al. (2010) reported significant differences at several EEG locations using the fractal dimension algorithms proposed by Higuchi and Katz. Finally, a statistically significant reduction in Lempel-Ziv complexity was found in ASD group in comparison with controls, at EEG electrodes F7, F3, and T5 (Sheikhani et al., 2007). All of these previous results are fully compatible with our findings of reduced AIS, as a reduced entropy limits also the maximally possible AIS. In addition, the findings of reduced Lempel-Ziv complexity align well with decreased AIS, as the Lempel-Ziv algorithm entails cataloguing recurrent events and this is tightly linked to predictable recurrence inside the repeated sequences in the signals. Our results extend these previous findings by localizing the dominant changes to the hippocampus. Moreover, the use of AIS, rather than more generic measures of entropy or complexity allows a straightforward interpretation in terms of component processes of information processing, i.e., information storage.

## 5. Conclusion

In this study, we present the first application of information theoretic measures of information storage to experimental neural data. Using MEG and source signal reconstruction from 12 selected brain areas, we show that AIS is reduced in the hippocampus of individuals with ASD. Future studies on larger samples of patients, combined with whole brain analyses, will have to show in how far our results generalize across brain areas, and to broader populations of ASD patients. The relatively uniform sign of the observed AIS differences across all investigated brain areas suggests that reduced AIS may be a pervasive change of information processing in ASD.

### Conflict of interest statement

The authors declare that the research was conducted in the absence of any commercial or financial relationships that could be construed as a potential conflict of interest.
